# Exercise, Nutrition and GLP-1 Medicines to Improve Body Weight and Muscle Function in Heart Failure with Preserved Ejection Fraction

**DOI:** 10.1007/s11897-026-00780-x

**Published:** 2026-10-08

**Authors:** Konstantinos Prokopidis, Aniket Sunil Joshi, Soren Gam, Kristina Norman, Ursula Müller-Werdan, Wolfram Doehner, Henning Tim Langer

**Affiliations:** 1 Department of Musculoskeletal Ageing and Science, Institute of Life Course and Medical Sciences, University of Liverpool, Liverpool, UK; 2 Department of Geriatrics and Medical Gerontology, Charité– Universitätsmedizin Berlin, Corporate Member of Freie Universität Berlin and Humboldt-Universität zu Berlin, Hindenburgdamm 30, Berlin, 12203 Germany; 3 Department of Endocrinology, Esbjerg Hospital, University Hospital of Southern Denmark, Esbjerg, Denmark; 4 Department of Sports Science and Clinical Biomechanics, University of Southern Denmark, Odense, Denmark; 5 Department of Regional Health Research, University of Southern Denmark, Odense, Denmark; 6 Department of Nutrition and Gerontology, German Institute of Human Nutrition Potsdam Rehbrücke, Nuthetal, Germany; 7 Berlin Institute of Health Center for Regenerative Therapies (BCRT), Charité-Universitätsmedizin Berlin, Berlin, Germany; 8 Deutsches Herzzentrum der Charité, Department of Cardiology, Angiology and Intensive Care Medicine, Berlin, Germany; 9 German Centre for Cardiovascular Research (DZHK), Partner Site Berlin, Berlin, Germany

**Keywords:** Heart failure, HFpEF, GLP-1 receptor agonists, Sarcopenia, Obesity, Physical function

## Abstract

**Purpose of Review:**

This narrative review synthesizes current evidence on lifestyle, pharmacological, surgical, and multimodal interventions targeting muscle strength, body composition, and functional capacity in HFpEF with sarcopenia and/or obesity.

**Recent Findings:**

Exercise, particularly concurrent endurance and resistance training protocols, improve aerobic capacity, functional performance, symptoms, and quality of life in HFpEF. Protein supplementation may preserve muscle mass, while caloric restriction may enhance aerobic capacity and function butdecreases muscle mass. SGLT2i and GLP-1 medicines may reduce hospitalizations and improve symptoms, with GLP-1 medicines producing greater weight loss and performance benefits. Bariatric surgery may also achieve substantial weight loss and functional improvements, although evidence in HFpEF with obesity is limited.

**Summary:**

Obesity-related HFpEF may benefit from a combination of resistance exercise and adequate protein intake to preserve muscle during weight loss, while pharmacotherapy can be used to support cardiac function. Future trials should evaluate multimodal interventions to mitigate frailty risk in HFpEF.

## Introduction

Age-related reductions in muscle mass and strength, clinically defined as sarcopenia [1], play a pivotal role in the pathophysiology and progression of heart failure with preserved ejection fraction (HFpEF). HFpEF is characterized by left ventricular ejection fraction (LVEF) of > 50% accompanied by diastolic dysfunction [2], while sarcopenia is driven by ageing-related processes such as anabolic resistance, increased protein degradation, mitochondrial dysfunction, and chronic low-grade inflammation, among others [3]. These processes may be further exacerbated by obesity through excess adiposity, systemic inflammation, metabolic dysregulation, and impaired mobility [4–6]. In HFpEF, these skeletal muscle-biased mechanisms may coincide with cardiac abnormalities, exacerbating exercise intolerance [7], and contributing to poor clinical outcomes [8], so much so that it has been suggested to redefine heart failure subtypes according to skeletal muscle mass [9]. The prevalence of sarcopenia in HFpEF is between 15 and 22% [10], though estimates may vary due to diagnostic criteria, population differences, and study settings: European and Asian cohorts report higher rates (31 and 35%, respectively), while American cohorts show lower figures (25%). Systemic factors, including reduced peripheral perfusion, low-grade inflammation, insulin resistance, and neurohormonal activation (e.g., elevated aldosterone, angiotensin, and catecholamines), could promote skeletal muscle catabolism *via* the ubiquitin-proteasome system, autophagy, and myostatin upregulation [11], which may be precursors of decreased lean soft tissue, strength, and overall quality of life [12]. In addition, reduced muscle capillarity, muscle fibre-type shifts (favouring glycolytic over oxidative fibres), and endothelial dysfunction may amplify exercise intolerance [11, 13]. A phenotype more commonly observed in HFpEF involves sarcopenic obesity, the coexistence of sarcopenia with excess adiposity [14]. Although its precise prevalence in HFpEF has yet to be established, considering that obesity is a prominent predictor of HFpEF, and that HF itself begets muscle wasting [11], the combination of these two could accelerate HFpEF progression [15]. Mechanisms behind sarcopenic obesity include dysregulated adipokine signaling, increased visceral and epicardial adipose tissue, and microvascular endothelial dysfunction, which could exacerbate myocardial stiffness, diastolic impairment, and insulin resistance [16]. At present, structured exercise training (particularly in the form of resistance exercise), nutritional interventions (e.g., low-calorie diets, protein supplementation), and pharmacological as well as surgical weight loss strategies (i.e., glucagon like peptide-1 (GLP-1) medicines, bariatric surgery), have attempted to optimize body compositions and enhance functional capacity in individuals with HFpEF, sarcopenia and/or obesity. The ideal effect of such treatments would be the reduction of excessive body weight, predominantly by reduction of adipose tissue with concomitant preservation of muscle mass and performance. In this narrative review, we aim to explore the evidence behind these interventions and identify research gaps that could lead to improved patient outcomes.

### Exercise Interventions

One of the first studies to investigate concurrent training (combined endurance and resistance exercise) in HFpEF was the Ex-DHF-P trial. Compared with usual care, three months of exercise improved physical function, health-related quality of life, and vitality in older, obese patients with HFpEF (65 ± 7 years) [17]. In a recent study, concurrent training for 12-weeks improved peak oxygen capacity (VO_2_peak) and Kansas City Cardiomyopathy Questionnaire (KCCQ) scores vs. non-supervised exercise recommendations in obese HFpEF patients with a mean age of 75.1 ± 7.2 years [18]. In a non-randomized controlled trial of slightly overweight, Japanese patients (aged 65–80 years), five months of supervised concurrent training three times weekly resulted in a better preservation of VO₂peak compared with usual care, where the control group lost ~ 12% while the exercise group modestly improved their values (+ 8%). However, only 30% of participants achieved a clinically meaningful improvement, defined as an increase of ≥ 3.0 mL/kg/min or ≥ 10% in VO₂peak [19]. In the largest randomized trial to date, 322 patients with HFpEF underwent 12 months of supervised concurrent training (three sessions/week combining endurance and resistance exercise). The intervention significantly improved VO₂peak and NYHA functional class compared with usual care. However, the between-group improvement in VO₂peak was modest (1.3 mL·kg⁻¹·min⁻¹) despite the prolonged intervention and relatively high training volume [20].

A secondary analysis of the HEART Camp randomized controlled trial evaluated an 18-month behavioural intervention designed to improve adherence to moderate-intensity aerobic exercise (40–80% heart rate reserve) and resistance training (10–15 repetitions per set to volitional fatigue) in 59 patients with HFpEF (mean age 64.6 ± 9.3 years; BMI 35.6 ± 7.2 kg/m²). Compared with optimized usual care, the intervention improved 6-minute walk distance by approximately 63 m (+ 16.5% vs. +3.7% in controls) and increased KCCQ clinical summary score by 9.8 points (+ 13.3%), whereas scores declined by 2.8 points (-3.8%) in the usual care group [21]. However, it is worth noting that adherence in the whole cohort, irrespective of heart failure phenotype, was modest, with 42% at month 12, and 35% at month 18 [22], indicating a need for further work to explore strategies that could promote greater exercise adherence.

A recent network meta-analysis including 13 randomized controlled trials (869 participants) ranked high-intensity interval training (HIIT) as the most effective lifestyle intervention for improving VO₂peak (+ 2.88 mL·kg¹·min⁻¹) and quality of life (MLHFQ) among the exercise- and dietary interventions evaluated. However, even the highest-ranked intervention produced only a modest improvement in VO₂peak, suggesting that exercise alone may have limited capacity to substantially improve cardiorespiratory fitness in patients with HFpEF [23]. The relatively modest improvements in VO₂peak across exercise interventions may partly reflect the fact that exercise intolerance in HFpEF is driven not only by cardiac dysfunction, but also by peripheral skeletal muscle abnormalities, including reduced muscle mass and quality, mitochondrial dysfunction, impaired capillary density, increased intramuscular fat, and impaired oxygen extraction [24].

### Nutritional Interventions

Older adults frequently consume insufficient amounts of protein, even when obese, with a higher BMI associated with a greater prevalence of inadequate intake [25] and in patients with heart failure specifically, lower estimated protein intake has been associated with increased mortality [26]. Consequently, a nutritional intervention reduced all-cause mortality and readmission due to worsening of heart failure (27% vs. 61%; HR 0.45, 95% CI 0.19–0.62; *p* = 0.0004) in hospitalised patients with malnutrition [27]. In older patients with HFpEF in general, the most direct rationale for protein supplementation is to simply correct the insufficient intake and prevent sarcopenia [28]. Protein or essential amino acid (EAA) supplementation may also counteract the anabolic resistance characteristic of heart failure that is a combined effect of a blunted response to anabolic signals (insulin resistance, growth hormone resistance) and increased catabolism [29]. A randomized crossover study in older women with heart failure showed that a beverage high in EAA indeed produced a greater net gain in whole-body protein than a higher-calorie, lower-EAA beverage [30].

Evidence for the effects of nutritional intervention or protein supplementation on functional outcomes in patients with HFpEF is, however, sparse. In a small study with older adults with HFpEF and obesity aged 65–75 years, protein supplementation alone (whey protein of up to 50 g/day) did not improve 6-minute walking distance (6MWD), stair climbing test, or gait speed vs. control (no intervention), but led to higher handgrip and quadriceps strength [31]. Given the very small sample size (~ 5–6 participants per arm), these findings are exploratory. Whether protein supplementation influences walking and gait-related outcomes in HFpEF remains uncertain.

The evidence for the effect of caloric restriction (CR) on functional outcomes in patients with obesity and HFpEF is stronger, though still resting on one trial. In this randomized, attention-controlled 2 × 2 factorial trial of 100 older, severely obese patients with HFpEF (mean age 67 years), 20 weeks of caloric restriction increased VO2peak by 1.3 mL/kg/min, an effect comparable to aerobic exercise training (1.2 mL/kg/min) and additive when combined [32]. CR alone also improved 6MWD, exercise time to exhaustion and peak workload, as well as producing a clinically meaningful improvement in disease-specific quality of life on the KCCQ, but not the MLHF score. One limitation of caloric restriction is loss of skeletal muscle mass which occurred despite adequate protein intake. CR alone significantly reduced DXA-measured lean mass (-2 kg; *p* < 0.0001) as well as MRI-measured thigh muscle area (-6 cm²; *p* < 0.0001). These findings suggest that caloric restriction and exercise training may improve exercise capacity through partly distinct but complementary mechanisms (i.e., weight loss or improved aerobic conditioning), supporting the use of combined interventions in obese patients with HFpEF.

### Pharmacological Interventions

Over the last 5 years, exciting new therapies for the treatment of HFpEF have emerged. While there are now multiple options for patients available that include diuretics, angiotensin receptor-neprilysin inhibitors, mineralocorticoid receptor antagonists (MRA), nonsteroidal MRA or angiotensin II receptor blockers, here we want to focus on sodium-glucose cotransporter 2 inhibitors (SGLT2i) and GLP-1 medicines, since both have been approved for treating metabolic diseases such as type II diabetes and/or obesity before they were approved for HFpEF. SGLT2i like empagliflozin [33] or dapagliflozin [34] have shown robust reductions of hospitalization and cardiovascular death in heart failure patients, leading them to be in the highest class of recommendation with the highest level of evidence by the European Society of Cardiology [35].

Similarly, GLP-1 medicines like semaglutide [36] and tirzepatide [37] have shown benefits such as reduced HFPEF symptoms and physical limitations, including improved exercise capacity (i.e., 6MWD). Interestingly, both therapies have additive effects, meaning that combining SGLT2i treatment with GLP-1 medicines results in superior heart protection compared to each of the drugs on their own [38]. This is likely related to the fact that the drugs exhibit their effects through distinct mechanisms: Even though both share anti-diabetic effects and cause weight loss, the physiology and extent of these vary robustly between treatments. For example, SGLT2i blocks the reabsorption of glucose into the blood stream through the kidneys. GLP-1 medicines on the other hand, predominantly reduce circulating glucose through increased insulin secretion by the pancreas. SGLT2 receptors do not appear to be expressed in the heart, and while the exact mechanisms of cardioprotection remain the focus on ongoing investigations, diuresis and systemic anti-inflammatory effects of SGLT2i likely contribute. At the same time, a modest weight loss is observed with SGLT2i in the range of 1–2 kg, largely resulting from volume shifts [39].

For GLP-1 medicines, in turn, a prominent weight loss in HFpEF patients with obesity has been observed, reaching anywhere between 10 and 15 kg [36, 37]. Notably, the GLP-1 receptor appears to be prominently expressed in cardiac tissue, and the cardioprotective effects of GLP-1 medicines are likely to be the result of direct myocardial signalling as well as indirect effects of the weight loss. Interestingly, despite the cardioprotection, SGLT2i’s cause only very mild functional improvements using the 6MWT, with a meta-analysis reporting an 11 m improvement in HFpEF patients [40]. For GLP-1RA semaglutide, the STEP-HFpEF trial reported an improvement of 22 m [36] and for tirzepatide the SUMMIT trial reported an improvement of 26 m [37] walking distance. Importantly, all of these effects on 6MWD are well below the proposed threshold of ~ 45 m to become clinically meaningful [41], while structured exercise programs have shown up to 50 m in individual trials and ~ 30 m across meta-analyses [42, 43], highlighting the value of multifactorial lifestyle interventions and the great potential for combined treatments and multi-modal therapy. Together, these data indicate that for SGLT2i’s and GLP-1 medicines, the reported cardioprotective outcomes do not necessarily correspond to an improvement in physical function as assessed via the 6MWT. However, GLP-1 medicines appear to have more pronounced effects on physical performance in clinically obese HFpEF patients who experience significant body weight loss [35, 36].

Aside from these observations, it needs to be emphasized that the weight loss effects of GLP-1 medicines must be treated carefully, particularly in older patients with HFpEF. While reductions in muscle size seem to be largely a function of weight loss with GLP-1 medicines [44], clinicians and dieticians need to be aware of the risk of sarcopenia and cachexia in older patients who often do not benefit from substantial weight loss, as it may increase frailty, hospitalizations and eventually mortality [45]. This needs to be considered for weight management recommendations with lifestyle interventions as well as medical treatment. Generally, current epidemiological evidence in older patients (70 years and beyond) would suggest that robust weight loss (equivalent to high dosing of GLP-1 medicines) should be reserved for morbidly obese patients with a BMI of > 35, while old patients who are just slightly overweight or mildly obese (BMI 25–30) are likely to benefit less from interventions that emphasize weight loss [46].

### Surgical Weight Loss Interventions

While the benefits outlined above may partly reflect direct cardiometabolic effects of GLP-1 medicines, the substantial weight loss achieved with these therapies is also likely to contribute. A separate, independent strategy to achieve weight loss is bariatric surgery. Evidence from bariatric surgery studies suggests that large reductions in body weight (~ 25%) can improve maximal walking speed by 6–9%, endurance capacity by ~ 10%, and sit-to-stand (STS) performance by ~ 9%, despite concomitant reductions in lean mass and absolute muscle power of 10–17% [47]. These findings indicate that weight loss itself may be an important driver of improved physical function in very obese individuals. Surgical weight loss has been shown to improve survival, lower hospitalization and decrease atrial fibrillation in obese patients with HFpEF, with the observed effects being equal or superior to pharmacological weight loss through GLP-1 medicines [48]. These results are echoed by broader investigations into obesity related heart failure, where several studies have suggested improvements in heart failure symptoms, quality of life, cardiac remodelling, and diastolic function following substantial weight loss via bariatric surgery [49]. Similarly, retrospective data in HFrEF patients with obesity suggests significant reductions in BMI after bariatric surgery, which was associated with improvements in LVEF, hospitalizations and mortality [24]. This highlights the potential of surgical weight loss in patients with varying types of heart failure (i.e., HFpEF as well as HFrEF). Unfortunately, original data on physical function in HFpEF patients following bariatric surgery are sparse. One study has investigated the effect of weight loss surgery on the Minnesota Living with Heart Failure Questionnaire (MLWHF), which includes questions related to physical function [50]. Patients in this study lost ~ 30% of their body weight and lowered their MLWHF score from ~ 27 to ~ 7, which was associated with improved diastolic relaxation, smaller left ventricular mass, improved blood lipids, decreased liver fat, lowered NYHA scores (from 3 to 4 to 1) and fewer medications required.

Data from obese patients without diagnosed heart failure supports substantial improvements in 6MWD with bariatric surgery: 49 patients reduced their BMI from ~ 51 to ~ 28 kg/m^2^ and improved their 6MWD by ~ 86 m [51]. Similarly, a study in 25 patients undergoing surgery reported a BMI reduction from ~ 46 to ~ 30 kg/m^2^ and a concomitant improvement of ~ 138 m in their 6MWD at 6 months post-surgery [52]. While both studies were not performed in patients diagnosed with HFpEF, these improvements in 6MWD after bariatric surgery are well above the proposed threshold of ~ 45 m to achieve a clinically meaningful improvement in physical function [41]. Interestingly, improvements in physical function following bariatric surgery do not necessarily coincide with preservation of lean body mass or muscle function (i.e., absolute strength and power). Recent data indicate that despite approximately 15% reductions in lean body mass and 12–20% reductions in muscle strength and power, patients improved maximal walking speed by 6–9%, endurance capacity by ~ 10%, and STS performance by ~ 9% following bariatric surgery [47]. While reductions in body weight alone may substantially improve functional capacity, the accompanying losses in lean body mass and muscle function may still be concerning in patients who already have low muscle mass or impaired muscle strength prior to surgery. Importantly, resistance exercise training has been shown to attenuate declines in muscle strength following bariatric surgery [53] and may therefore be a valuable adjunct for vulnerable patients undergoing surgical weight loss interventions. Future studies need to more directly explore the potential of weight loss surgeries to target mobility in patients with HFpEF, as an alternative or combined approach to lifestyle and pharmacological interventions.

### Combined Interventions

**Fig. 1:**
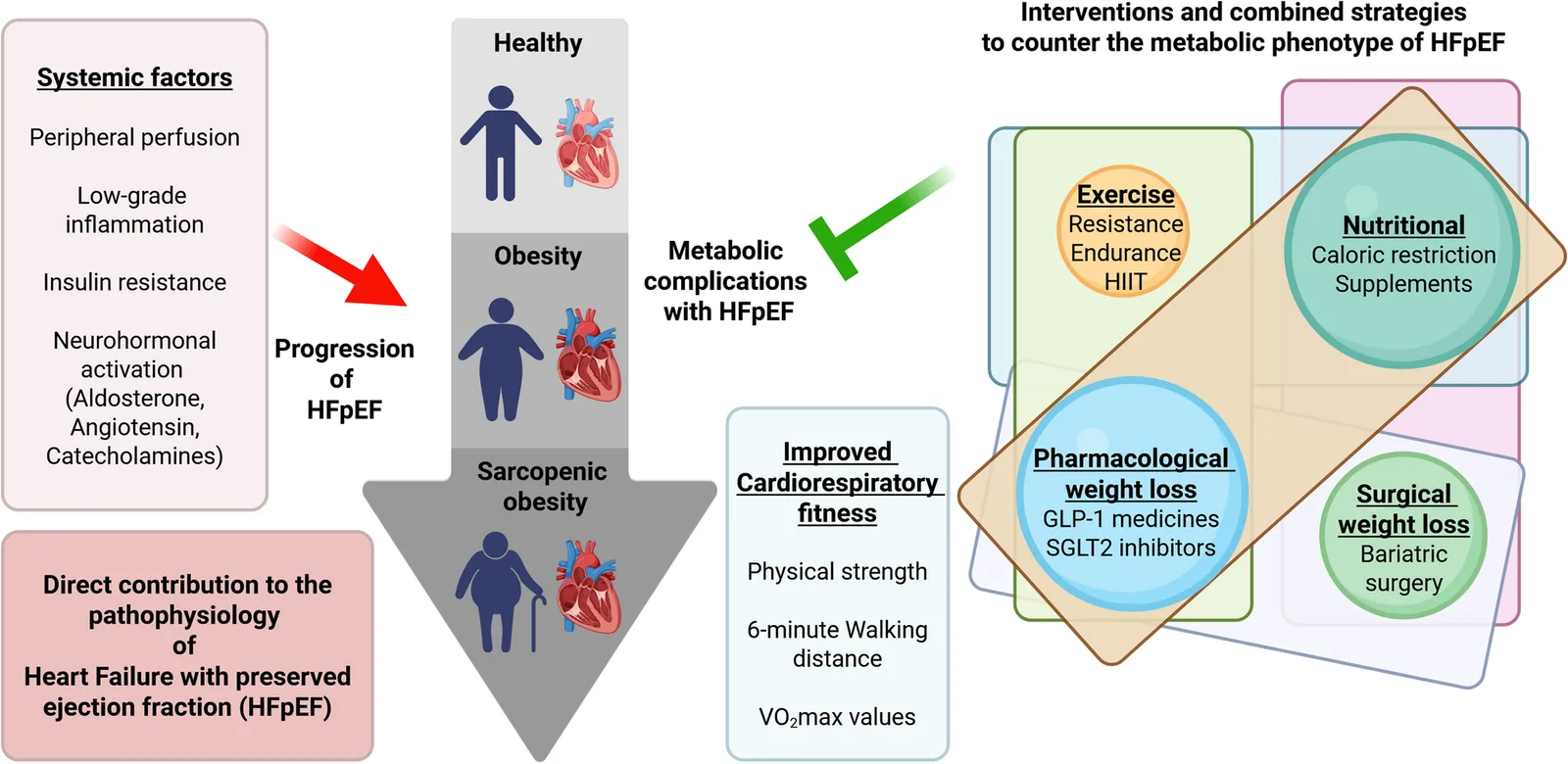
Graphical representation of the interaction between systemic factors driving HFpEF disease progression, pharmacological treatments to attenuate the disease, and various individual or combined strategies to target an improvement of the metabolic phenotype and physical function in HFpEF patients, to avoid the deterioration into sarcopenic obesity and HFrEF

Acute caloric restriction, when combined with exercise, has been shown to restore muscle growth in rats with obesity and HFpEF, by increasing myonuclear accretion and restoring myogenic homeostasis [54], suggesting that combined calorie restriction and exercise approaches may be particularly suitable for improving skeletal muscle pathology in HFpEF. In humans with HFpEF, concurrent training (i.e., resistance-type exercise and endurance exercise) combined with caloric restriction compared to just aerobic training with caloric restriction, exhibited significantly greater increases in leg muscle strength and leg muscle quality, while both regimes enhanced VO_2_peak, KCCQ, LV mass, and arterial stiffness, and demonstrated similar changes in body weight, body fat, and skeletal muscle mass after 20 weeks [55]. Similarly, resistance training (2x/week for 12-weeks) combined with whey protein (up to 50 g/day), led to greater improvements of 6MWD, stair climbing test, timed up and go, gait speed, handgrip and quadriceps strength than whey protein alone and a non-intervention control [31], underscoring the positive impact of exercise on functional capacity. Currently, a prospective, randomised, controlled clinical trial in patients with HFpEF and sarcopenia explores the impact of concurrent training (aerobic training 2x/week for 40–50 min; resistance training 2x/week with 40–70% one-repetition maximum for 10 repetitions) combined with whey protein vs. resistance training alone vs. control on muscle mass, 6MWD, cardiac function, and quality of life for 12 weeks [56]. However, despite the great potential highlighted in this review, clinical trial data on HFpEF patients undergoing a combined treatment of lifestyle intervention such as exercise training and pharmacological- or surgical weight loss is not yet available. The interaction between the disease progression, medical treatment, the metabolic phenotype and the individual and combined treatments listed above are summarized in (Fig. 1).

## Conclusions

Given the high prevalence of sarcopenia and obesity in patients with HFpEF, all three indications are strongly interwoven. Anabolic resistance, excess adiposity and decreased muscle mass and function, resulting in very low physical performance are the most common hallmarks. The low exercise capacity can be primarily counteracted through lifestyle interventions that involve physical activity, while GLP-1 medicines and bariatric surgery offer highly efficient methods to substantially reduce bodyweight in patients. Nutritional interventions such as high protein intake can help preserve muscle mass during weight loss and SGLT2i/GLP-1 medicines and other emerging drugs can directly ameliorate heart failure symptoms and improve survival in a weight loss independent manner. Interestingly, for each treatment individually, the available data on the effects on physical function in HFpEF patients are small to modest. While RCT data on combinational treatments are sparse, we think that circumstantial evidence strongly favours multimodal interventions for HFpEF patients: Direct treatment of the heart failure through pharmacological means should be tightly connected to exercise interventions that target physical function,as well as caloric restriction through lifestyle modifications, incretin mimetics or surgical strategies to induce weight loss (if excess adiposity is present), and nutritional interventions like protein supplementation to help maintain muscle mass during weight loss. Cardiologists, exercise physiologists and nutritionists are strongly encouraged to explore these avenues in future clinical trials and practice.

Finally, practitioners also need to be careful not to overemphasize weight loss as a target in very old patient populations (> 65 y) without adequately supporting the maintenance of muscle mass. We have previously commented on the risk of cardiac cachexia [46] and accelerated sarcopenia [45] in aged populations undergoing substantial weight loss. Epidemiological evidence consistently supports the notion that weight loss and weight fluctuations increase mortality in older populations [57], and we know that cardiac cachexia is a highly prevalent complication in heart failure [58], which increases mortality. As such the preservation of muscle mass through a combined application of weight loss interventions with resistance exercise and increased protein intake becomes not only a superior treatment strategy in this population (i.e., aged and HFpEF) but an urgent necessity. In the absence of approved pharmacological solutions for the maintenance of muscle mass and function, exercise and nutrition are currently the only available bona fide treatment for such patients.

## Data Availability

No datasets were generated or analysed during the current study.

## Key References

- Prokopidis K, Irlik K, Hendel M, Piaśnik J, Lip GY, Nabrdalik K. Prognostic Impact and Prevalence of Cachexia in Patients With Heart Failure: A Systematic Review and Meta-Analysis. Journal of cachexia, sarcopenia and muscle. 2024;15(6):2536-43. 
  - ○ This meta-analysis found that loss of muscle mass and strength are highly prevalent in patients with heart failure and independently predict all-cause mortality.
- Shoemaker MJ, Curtis AB, Vangsnes E, Dickinson MG. Triangulating clinically meaningful change in the six-minute walk test in individuals with chronic heart failure: a systematic review. Cardiopulmonary physical therapy journal. 2012;23(3):5. 
  - ○ Here, the authors suggest 45 m as a threshold for clinically meaningful improvements in the 6-minute walking test.
- Edelmann F, Wachter R, Duvinage A, Mueller S, Fegers-Wustrow I, Schwarz S, et al. Combined endurance and resistance exercise training in heart failure with preserved ejection fraction: a randomized controlled trial. Nature Medicine. 2025;31(1):306−14. 
  - ○ This study found improvements in VO2 and NYHA class with one year of concurrent exercise training in HFpEF patients.
- Kosiborod MN, Abildstrøm SZ, Borlaug BA, Butler J, Rasmussen S, Davies M, et al. Semaglutide in patients with heart failure with preserved ejection fraction and obesity. New England Journal of Medicine. 2023;389(12):1069-84.
  - ○ This large clinical trial reported improvements in symptoms, body weight and physical performance of HFpEF patients treated with the GLP-1RA semaglutide.
- Gam S, Hermann AP, Juhl CB, Hansen SG, Gram B. Effect of Zoledronic Acid on Skeletal Muscle After Bariatric Surgery: A Secondary Analysis From a Randomized Controlled Trial. Obesity. 2026;34(1):76–87.
  - ○ This manuscript highlights the positive effects of substantial weight loss (through surgery) on physical performance.
